# Soldier's Heart

**Published:** 1911-04-22

**Authors:** 


### SOLDIER'S HEART.

The condition known as "soldier's heart" has been a subject of military medical interest for many years. It was at one time thought to be largely due to the tight unsuitable tunic, laced by cross straps in front and behind in such a way as to impede expansion of the chest during exertion, and this belief was largely responsible for the abolition of this type of uniform. Nevertheless it has been found that, in spite of the undoubted improvement, a certain percentage of cases still occurs in our Army, and in a recent number of the *Journal of the Royal Army Medical Corps*, Lieut.-Colonel R. S. Simpson reviews the subject from the standpoint of comparative statistics. In 1889 the number of recruits rejected for valvular diseases of the heart was 22 per 1,000, but in 1900, during the rush of the South African war, it fell to 12 per 1,000, to rise to 20 per 1,000 again in 1903. Since 1904 there has been an apparent stiffening in the standard of examination, for the proportion of rejections rose to 26 per 1,000 in 1905, 32 per 1,000 in 1906, and 34 per 1,000 in 1908.

Such being the case, one would naturally expect that as the recruits admitted were sounder as regards their hearts, there ought to have been a proportionate diminution in the number of cases admitted to hospital or discharged from the service for soldier's heart. This unfortunately has not been the case. The average number of admissions for this condition in the British Army is 4 per 1,000. Between 1900 and 1903 it rose as high as 10 per 1,000, probably due to the reduced standard, as well as the effects of the Boer war. This rise may have been responsible for the stiffening of the standard in 1904. The results have been that although the percentage of cases has again sunk to the average of 4 per 1,000 there has been no further diminution, in spite of the theoretically improved physical standard of the admitted recruits.

The condition seems to occur most frequently in men between twenty and twenty-five years of age, and the highest curve appears about the third year of service. The clinical features noted are: impairment of circulation as shown by cold-blue hands and feet, and general loss of tone; increased reflexes, especially in the arms; a variable pulse-rate, easily accelerated by examination; a high blood-pressure up to 140 mm. of mercury, which may rise or fall after exercise; no heart murmurs as a rule, and faintness and giddiness on parade. Treatment in bed or in hospital seems to make the patients worse. Graduated exercises and hot baths appear to give the best results. Tonics are useless. The prognosis as regards further military service is bad; and prevention is probably impossible, as the condition seems to be congenital or at least developmental. It can, however, be aggravated by unsuitable clothing, and a wide collar is useless if the tunic is so badly made that the collar is pulled down and pressed on the neck below the larynx.

